# The Role of *Liriodendron Dof* Gene Family in Abiotic Stress Response

**DOI:** 10.3390/plants13142009

**Published:** 2024-07-22

**Authors:** Bojun Liao, Pengxiang Liang, Lu Tong, Lu Lu, Ye Lu, Renhua Zheng, Xueyan Zheng, Jinhui Chen, Zhaodong Hao

**Affiliations:** 1 State Key Laboratory of Tree Genetics and Breeding, Co-Innovation Center for Sustainable Forestry in Southern China, Nanjing Forestry University, Nanjing 210037, China; 15779776739@163.com (B.L.); lpx20021@163.com (P.L.); tonglu202402@163.com (L.T.); lulu2020@njfu.edu.cn (L.L.); luye@njfu.edu.cn (Y.L.); 2 Key Laboratory of Forest Genetics & Biotechnology of Ministry of Education, Nanjing Forestry University, Nanjing 210037, China; 3 Fujian Academy of Forestry, Fuzhou 350012, China; zrh08@126.com (R.Z.); zxy0553@163.com (X.Z.); 4 National Germplasm Bank of Chinese Fir at Fujian Yangkou Forest Farm, Shunchang, Nanping 353211, China

**Keywords:** DNA-binding with one finger, *Liriodendron*, abiotic stress

## Abstract

The DOF (DNA-binding with one finger) transcription factors are exclusive to plants and play crucial roles in plant growth, development, and environmental adaptation. Although extensive research has been conducted on the *Dof* gene family in *Arabidopsis*, *maize*, and *Solanum*, investigations concerning the role of this gene family in *Liriodendron* remain unreported, leaving its biological function largely unknown. In this study, we performed a comprehensive genome-wide identification of the *Dof* gene family based on the *Liriodendron* genome, resulting in the discovery of a total of 17 *LcDof* gene members. Based on the results of phylogenetic analysis, the 17 LcDof proteins were classified into eight subfamilies. The motif analysis revealed the diverse nature of motifs within the D1 subfamily, which includes a distinct type of Dof transcription factor known as *CDF* (Cycling Dof Factor). We further characterized the chromosomal distribution, gene structure, conserved protein motifs, and cis-elements in the promoter regions. Additionally, utilizing transcriptome data from *Liriodendron* hybrids and conducting RT-qPCR experiments, we investigated the expression patterns of *LhDofs* under various abiotic stresses such as drought, cold, and heat stress. Notably, we found that several *LhDofs*, particularly *LhDof4* and *LhDof6*, were significantly upregulated in response to abiotic stress. Furthermore, we cloned *LhDof4* and *LhDof6* genes and found that its encoding protein was mainly located in the nucleus by transient transformation in *Liriodendron* hybrids protoplast. Subsequently, we used *LhDof6*-overexpressing *Liriodendron* hybrid seedlings. We found that overexpression of *LhDof6* enhanced the cold tolerance of the plants, increasing their survival rate at −20 °C. This result was further validated by changes in physiological indicators.

## 1. Introduction

The presence of abiotic stresses, such as high salinity, drought, extreme temperatures, and poor soil fertility, poses significant environmental challenges that critically impede plant growth, development, and overall productivity [1]. Throughout the process of evolution, plants have demonstrated their ability to gradually colonize a wide range of terrestrial environments, including those characterized by harsh and extreme conditions. This remarkable adaptability has been facilitated by the development of sophisticated molecular and physiological mechanisms that enable plants to regulate their growth in response to resource availability and prevailing environmental conditions. The aforementioned adaptations have played a pivotal role in enabling plants to flourish in various ecosystems, ranging from arid deserts to saline marshes, thereby showcasing their remarkable resilience and adaptability in surmounting environmental constraints [1,2,3,4,5].

Numerous transcription factors (TFs) involved in the regulation of gene expression and signaling pathways related to abiotic stress have been identified, encompassing a diverse range of members from large gene families such as bHLH, HD-ZIP, WRKY, MYB, bZIP, DOF, and NAC [6,7,8,9,10].

The Plant-specific DNA Binding with One Finger (DOF) proteins are a group of transcription factors (TFs) characterized by a conserved 50-amino-acid DNA-binding domain, typically located in their N-terminal region and connected to a basic region [11]. The conserved DOF domain is a distinct zinc finger domain, characterized by a C2–C2 finger structure. It specifically binds to *cis*-regulatory DNA elements featuring the core 5′-T/AAAG-3′ motif, which is found in the promoter regions of target genes [12,13]. Recent studies have revealed that, despite its initial identification as a DNA-binding domain, the DOF domain may possess a plethora of functions, including nuclear localization, interaction with other transcription factors and intercellular trafficking [14,15]. Previous studies have corroborated its functional role in plant growth and development, including flowering control [16], maturation, seed development [17], and germination [18,19]. In particular, mutant dag1 (which encodes a Dof transcription factor in *Arabidopsis*) seeds are induced to germinate by significantly red light fluence rates [20]; the *COG1* gene (which encodes a Dof protein in *Arabidopsis*) functions as a negative regulator in phytochrome signaling pathways [21]. Additionally, compelling evidence suggests that CYCLING DOF FACTORS (*CDFs*), a class of Dof-type transcriptional repressors, have been experimentally proven to directly suppress the expression of CONSTANS (*CO*). *CDFs* possess the ability to inhibit the expression of photoperiodic genes, thereby influencing the perception of day length and ultimately impacting the floral transition in *Arabidopsis* [22]. More significantly, Dof transcription factors play a crucial role in plant phytohormone and stress responses. For instance, *TDDF1*, which encodes a Dof protein in tomato, enhances tolerance to drought, salt, various hormonal stresses, as well as resistance to late blight [23]. The salt and osmotic stress tolerance is enhanced by *ThZFP1* and *ThDof1.4* through the elevation of proline levels and improvement in ROS scavenging capability [24]. Therefore, the *Dof* gene family plays an essential role in the life cycle of plants.

The *Liriodendron* hybrids display significant heterosis, encompassing not only distinct foliar morphology and exotic floral characteristics but also notable adaptive capabilities and growth advantages. Historically, the *Liriodendron* hybrids have shown sensitivity to low-temperature stress, which has posed a considerable challenge. The study and functional validation of the *Dof* gene family may provide a potential avenue for identifying candidate genes that can be utilized for genetic improvement and the development of cold tolerance in *Liriodendron* hybrids. Furthermore, proposing further application of genetic engineering techniques for developing novel cold-resistant *Liriodendron* hybrids aims to extend these new varieties’ reach to facilitate ecosystem services across a broader geographical area.

## 2. Results

### 2.1. Identification and Protein Sequence Characterization of LcDofs

The *Dof* gene has been previously confirmed to be broadly involved in plant growth and development. Recent reports have also identified its significant role in plant responses to abiotic stress. The *Dof* gene in *Liriodendron chinense* (*L. chinense*) has been the subject of initial exploration and analysis. The *L. chinense* genome contains a total of 17 *Dof* genes, accounting for 0.048% of the overall gene count in the genome (Table 1).

The Dof transcription factor family in *L. chinense* is relatively small compared to the majority of species. Based on their chromosomal locations, these genes have been designated as *LhDof1* to *LhDof17* (Table 1). To investigate the genetic diversity within this family, we conducted a comprehensive analysis of the fundamental physicochemical characteristics of these 17 Dof proteins, encompassing protein sequence length, molecular weight (MW), isoelectric point (pI), and other pertinent properties. The analysis of the physicochemical properties of the *LcDof* gene family revealed that the Dof proteins exhibit a length range of 160 to 635 amino acids, with molecular weights varying from 17.01 to 71.60 kDa. The isoelectric point (pI) of LcDof7 (Lchi21078) was observed to be the lowest at 5.70, while the highest pI value of 9.56 was noted for LcDof5 (Lchi18955), with an average pI of 7.76. The analysis indicates that the majority of Dof proteins exhibit a weakly basic nature. Most LcDof proteins are characterized by high levels of instability, with the highest instability index score reaching 85.81. Among this group, only LcDof11 (Lchi08966) is considered stable, as it exhibits an instability index value below 40. The LcDof proteins are susceptible to denaturation or degradation, leading to potential alterations in their physicochemical properties and biological functions. This implies that LcDof proteins exhibit a high sensitivity towards changes in the external environment. The physicochemical properties of Dof proteins in *L. chinense* display variations, suggesting diverse regulatory roles in plant growth and development under different conditions. Therefore, it is crucial to conduct a comprehensive investigation into their classification and phylogenetic traits.

### 2.2. LcDofs Contain More Abundant Conserved Motifs and a More Homogeneous Gene Structure

**Figure 1:**
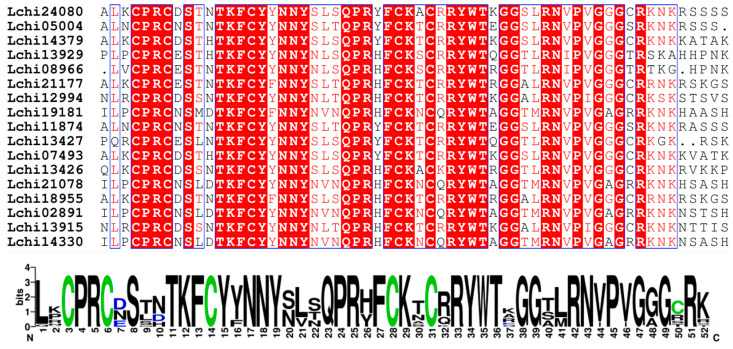
The Dof concerved region in LcDofs, alignment of multiple protein sequences in LcDofs.

**Figure 2:**
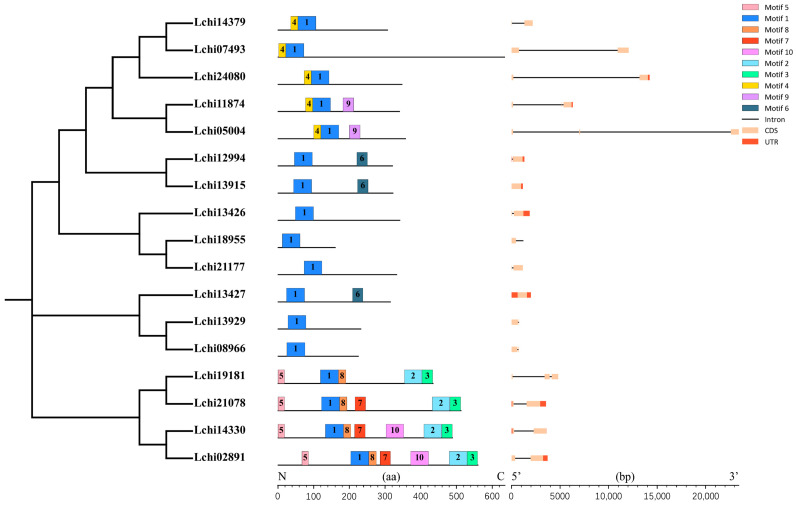
Analysis of conserved motif elements and gene structures of *LcDofs*. Figure was made using the MEME program and TBtools.

A multiple sequence alignment of 17 LcDof amino acid sequences from *L. chinense* was performed using the ClustalX software (V2.1). The conserved domains were then analyzed. It was found that the N-terminal Dof domain of the *L. chinense* Dof protein contains a typical C2–C2 zinc finger protein structure, comprising 52 amino acid residues (Figure 1). The LcDof protein domain remains intact and exhibits a remarkable level of uniformity, comprising a solitary zinc finger protein composed of four cysteines. This observation signifies the highly conserved and complete nature of the Dof domain in *L. chinense*. The *LcDof* gene family is classified into 6 subgroups based on multiple sequence alignment and phylogenetic analysis, with each subgroup exhibiting nearly identical motif structure and distribution of LcDof proteins (Figure 2). Moreover, genes within the same subgroup share analogous intron-exon structures and gene lengths (Figure 2). All LcDof proteins contain Motif1 (Figure 2), which is consistent with the findings of previous studies. Motif1 is the conserved Dof motif of LcDof. Our findings indicate that the C-terminal structures of four LcDof proteins (Lchi19181, Lchi21078, Lchi14330, Lchi02891) within a specific subgroup are characterized by the presence of diverse types of motifs. This observation suggests that the LcDof proteins in this subgroup may play a role in a multitude of biological processes. The presence of other motifs, such as Motif 4, 6, and 9, is limited to only a single or a few phylogeographic subgroups. Interestingly, in certain categories, no additional conserved sequence motifs were identified besides the Dof motif, implying that the functions of these sequences may be unpredictable. A comprehensive analysis of these sequences may prove crucial in elucidating the functional diversity of the Dof family. Through gene structure analysis, it was observed that the *LcDof* genes exhibit a distribution of 1 (2 genes), 2 (11 genes), and 3 (4 genes) exons, respectively, suggesting that the predominant form of *LcDof* genes consists of two exons.

### 2.3. Phylogenetic Structure of the LcDof Gene Family

**Figure 3:**
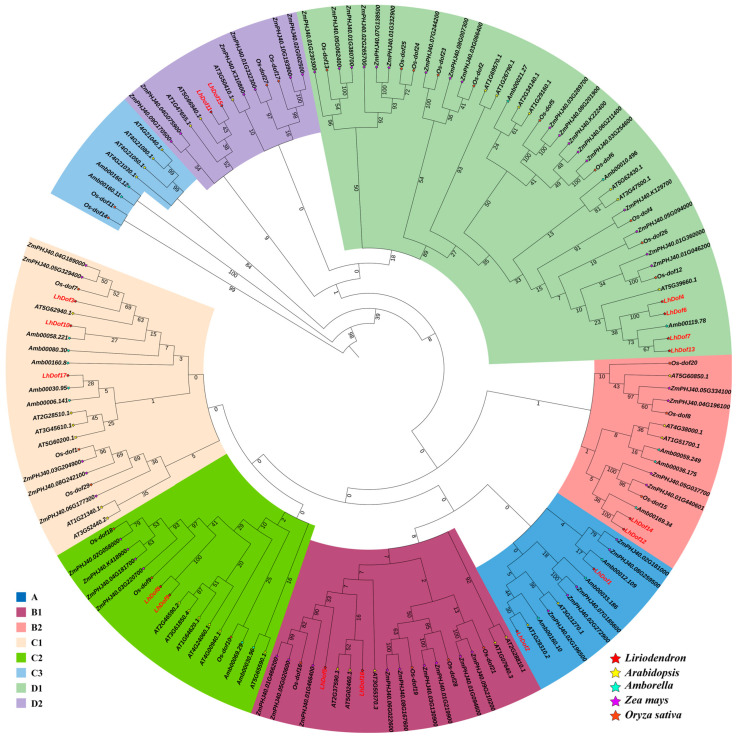
Phylogenetic trees of *Dof* genes. Different colors represent different subclasses, and the red font represent *L. chinense*. Numbers on branches indicate percent reliability of bootstrap values based on 1000 replicates.

In order to elucidate the molecular evolution and phylogenetic relationships of *L. chinense* Dof proteins, an unrooted phylogenetic tree was constructed containing 17 LcDofs and their homologs in *Arabidopsis thaliana*, *Amborella trichopoda*, *Zea mays*, and *Oryza sativa*. A multiple sequence alignment of *Dof* gene family members was performed using MAFFT software (v7.487) (https://mafft.cbrc.jp/alignment/software, accessed on 25 January 2022) with default parameters. The phylogenetic tree was constructed using MAGE 7.0, employing the neighbor-joining method with a bootstrap value of 1000 to analyze the evolution of the *Dof* gene in *L. chinense* (Figure 3). The results of multiple sequence alignment and phylogenetic tree clustering indicate that the Dof proteins from *Liriodendron chinense*, *Arabidopsis thaliana*, *Amborella trichopoda*, *Zea mays*, and *Oryza sativa* can be classified into eight distinct categories: Class A, B1, B2, C1, C2, C3, D1, and D2 (Figure 3). Additionally, *L. chinense* and *A. trichopoda* exhibit a closer phylogenetic relationship.

### 2.4. Analysis of the Whole-Genome Duplication Events in the LcDof Family

**Figure 4:**
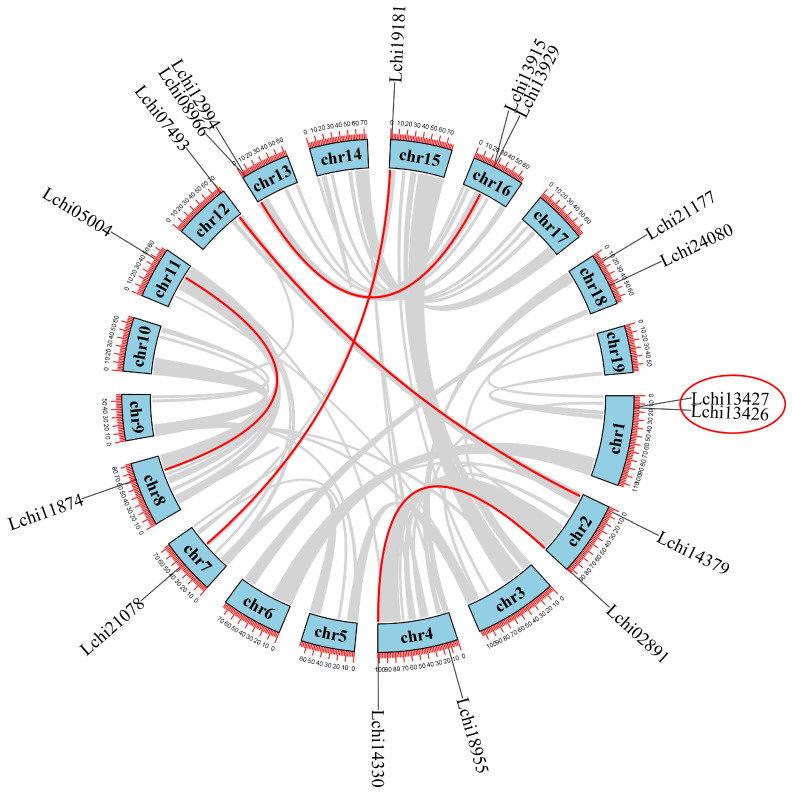
Genome-wide synteny analysis of *Dof* gene family among *L. chinense*. Distribution and duplication of the *LcDof* gene in *L. chinense*. The circles are chromosomal genes, and the names are displayed outside the circles.

In order to gain insight into the chromosomal distribution of the *LcDof* family, we used Tbtools software along with the genomic localization information of the *LcDof* family members to display their chromosomal distribution (Figure 4). The distribution of *LcDof* family members across the chromosomes is not uniform, with *Dof* genes found only on chromosomes 1, 2, 4, 7, 8, 11, 12, 13, 15, 16, and 18 in *L. chinense*. Among them, chromosomes 1, 2, 4, 13, 16, and 18 each contain two *Dof* genes, and the genes are closely arranged on chromosomes 1, 13, and 16. The data indicates that each chromosome contains a maximum of two *Dof* genes, suggesting that *LcDof* genes are typically spaced at considerable distances and rarely clustered on the same chromosome. The majority of *LcDof* genes are located at the chromosomal ends, with fewer near the centromeric regions. The concentrated distribution of *LcDof* genes at the chromosomal ends in *L. chinense* may be related to the more open chromatin structure found at these locations, which could facilitate active gene expression. Meanwhile, the chromosomal center (near the centromeres) typically has a more compact chromatin structure, which may restrict gene expression [25,26].

We have studied and mapped gene duplication events during the evolutionary process of *L. chinense*, which may also affect changes in the number and distribution of *LcDof* genes within the genome (Figure 4). It is known that duplication events occur regionally through a process known as tandem duplication, whereby gene sequences of less than 200 kb are copied in close proximity to the original gene. However, they can also occur over a broader range through segmental duplication, where larger fragments are duplicated. The former is typically attributed to DNA replication errors, whereas the latter may be attributed to polyploidy events resulting from chromosomal rearrangements [27,28]. There are six duplication events within the *LcDof* genes of *L. chinense* (Lchi21087–Lchi19181, Lchi05004–Lchi11874, Lchi14330–Lchi02891, Lchi07493–Lchi14379, Lchi08966–Lchi13929), of which only one pair (Lchi13427–Lchi13426) is due to tandem duplication, while the rest are caused by segmental duplication. The analysis of the expansion of the *LcDof* gene family has revealed that the majority of gene duplication events are the result of segmental duplication, which represents a crucial mechanism for gene expansion in plants. The presence of these duplication events reveals significant expansion strategies within the gene family during the evolutionary process and also indicates the dynamic changes and adaptive evolution of plant genomes. The aforementioned duplication pattern, in conjunction with the whole-genome duplication (WGD) event experienced by *L. chinense*, offers crucial insights into the expansion and adaptation of the *LcDof* gene family throughout plant evolution. Following whole-genome duplication events, some genes may be lost due to functional redundancy, while genes with essential functions or those providing adaptive advantages are often retained.

### 2.5. The Analysis of Cis-Element Regulation of Promoters Revealed That LcDofs Regulated Many Bioactive Processes

**Figure 5:**
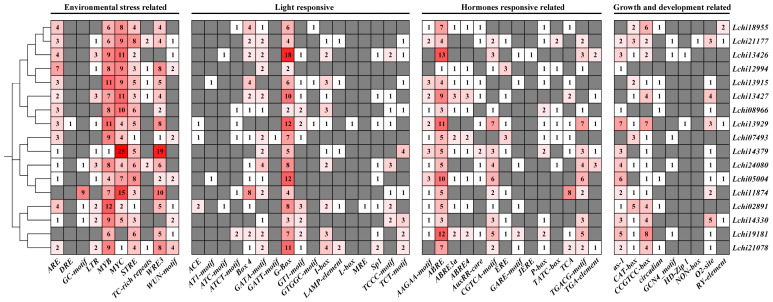
Results of *LcDofs* gene promoter predictor elements from the PlantCare database are depicted. The abscissa axis displays the types of elements. Red squares indicate a higher predicted presence of the element in the *LcDofs* gene promoter, while gray and white squares signify that the element is either absent in the gene promoter or present in lesser numbers.

We used the PlantCARE website to analyze the 5′ upstream promoter regions (2000 bp) of the *LcDof* genes to predict all cis-acting elements. A variety of cis-acting elements were identified, and the promoter regions of all *LcDof* genes were found to contain a substantial number of them. We classified these cis-acting elements into four categories: growth and development-related elements, light-responsive elements, abiotic stress-responsive elements, and plant hormone-responsive elements (Figure 5). The promoters of *LcDof* genes were found to be rich in light-responsive and abiotic stress-responsive elements, which suggests that *LcDof* genes are primarily involved in biological processes related to light response and abiotic stress response. In summary, the distribution of cis-acting elements indicates that *LcDofs* are involved in light response, hormone response, stress response, and plant growth and development.

The presence of MYB and STRE environmental response factors in all *LcDof* genes indicates that these two cis-acting elements are crucial for *LcDof* genes to perceive environmental alterations. However, some cis-acting elements are specific to certain *LcDofs*. For example, only *LcDof15* (Lchi13929) in Class D2 contains the DRE element, which suggests that Class D2 *LcDofs* may have differentiated to include proteins that are responsive to dehydration. The *LcDof* family members contain LTR functional elements, suggesting that some members may be specifically involved in cold response. While not all members within each class contain LTR elements, at least one member in each class is capable of responding to cold, indicating that the *LcDof* family exhibits some sensitivity to low temperatures.

G-Box, a crucial cis-regulatory element in light response, is abundant in all members of the *LcDof* family, playing a significant role in mediating light-dependent gene expression. The G-Box is commonly found in the promoters of many light-responsive genes, where it binds to light-activated transcription factors to promote transcriptional activation of these genes in response to light stimuli. Other light-responsive elements appear to be irregularly distributed in the promoters of each member, with *LcDof4* (Lchi02891) containing more light-responsive elements than other members, indicating differences in light response capabilities within the family. As *LcDofs* contain abundant ABRE elements, they may be actively involved in the abscisic acid response of plants. The distribution and quantity of ABRE3a and ABRE4 elements are evenly spread among specific *LcDof* members. This pattern suggests that these genes likely play a consistent and significant role in plant’s response to ABA signaling. The specific distribution of ABRE3a and ABRE4 elements may ensure the coordinated expression of these genes throughout the plant, effectively regulating the physiological state of the plant to adapt to environmental changes [29,30]. While ABRE elements are widely distributed in certain *LcDof* members, this does not imply that all members have identical functions in all physiological processes or stress responses. The different ABRE types may act as mediators for the binding of specific transcription factors or co-activators, which in turn result in subtle functional differences that enable plants to adapt their response to abscisic acid in a precise manner [31,32]. The results suggest that these LcDof proteins may also be involved in different abscisic acid-mediated regulatory networks. The majority of the growth and development regulatory elements of *LcDofs* are concentrated in the meristematic and differentiation functions of tissues, suggesting that these genes play a pivotal role in tissue differentiation and development.

The cis-regulatory element analysis reveals that the functional elements in the promoter regions of *LcDof* genes are both abundant and comprehensive, indicating that they may function independently or simultaneously to regulate growth, development, and abiotic stress response. Accordingly, further investigation is required to elucidate the expression profiles of *LcDofs* under growth conditions and abiotic stress.

### 2.6. LcDof Gene Families of Class D1 under Abiotic Stress Has a Strong Reaction

**Figure 6:**
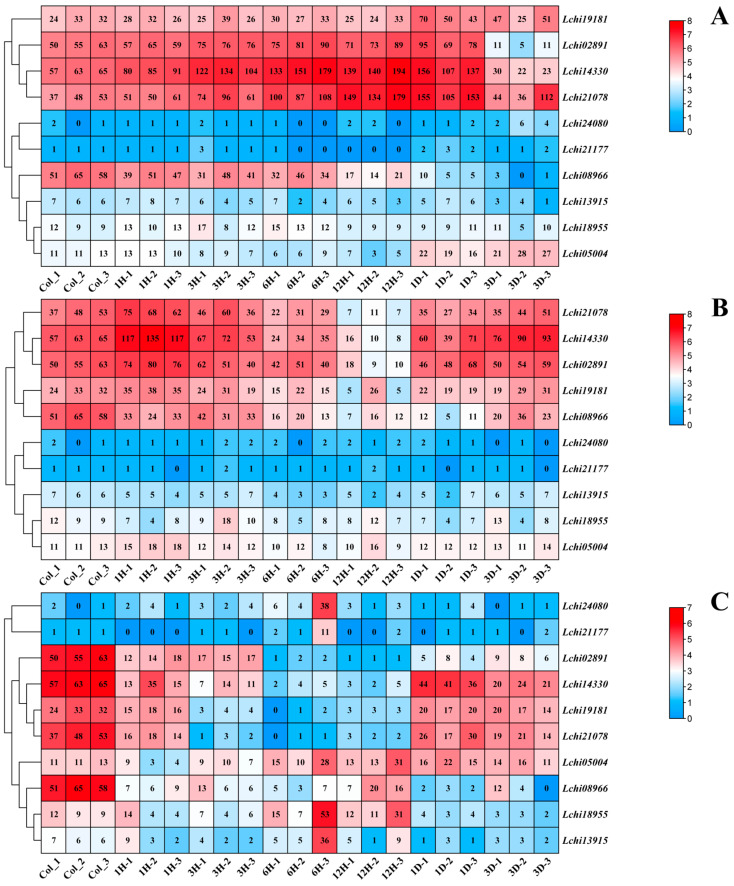
Transcriptional expression patterns of *Dof* genes in *L.* hybrid under (**A**) cold, (**B**) drought, and (**C**) heat stress are depicted. The *LhDofs* were subjected to three different stress factors: cold, drought, and heat stress. The designations Heat_0h, Heat_1h, Heat_3h, Heat_6h, Heat_12h, Heat_1d, and Heat_3d represent three biological replicates for each time point (0 h, 1 h, 3 h, 6 h, 12 h, 1 day, 3 days). Transcript abundance levels are represented using the log2 (FPKM + 1) transformation. The values on the right panel of the heatmap indicate the expression level.

To study the expression pattern of *Liriodendron* hybrid *Dof* genes under different stress conditions, we analyzed their expression profiles in the transcriptome data of leaves under low temperature, PEG6000-simulated drought, and high-temperature stress conditions (Figure 6). The results indicate that *LhDof* genes in the D1 class are actively expressed, and both *LhDof4* (Lchi02891) and *LhDof6* (Lchi14330) exhibit strong responses to cold and drought stress, with similar expression patterns (Figure 6A,B). Under low-temperature treatment, the expression levels of *LhDof4* and *LhDof6* were continuously upregulated from 0 h (CK) to 1 day, peaking at 12 h, and then downregulated from 1 day to 3 days (Figure 6A). Under PEG6000-simulated drought treatment, the expression levels of *LhDof4* and *LhDof6* were continuously upregulated from 0 h (CK) to 1 h, reaching their highest expression at 1 h, gradually downregulated from 1 h to 12 h, and then slowly upregulated from 12 h to 3 days, finally maintaining normal expression levels (Figure 6B).

The expression levels of *LhDof4* and *LhDof6* were significantly downregulated under high-temperature treatment. However, they exhibited high responsiveness to low-temperature and drought stress, displaying similar expression patterns across all three stress conditions (Figure 6C).

The D1 class of the *Dof* family encompasses a specific category of *Dof* genes, designated as *CDF* genes, which typically demonstrate robust responses to abiotic stressors. We have confirmed this in *Liriodendron* hybrid as well. The CDF transcription factors *LhDof4* and *LhDof6* from the D1 class may play a positive regulatory role under low-temperature and drought stress. The sharply downregulated expression of these two factors under high temperature stress suggests that *LhDof4* and *LhDof6* have a negative regulatory role in response to high temperatures.

Two of the *LcDof* genes were not expressed under the two stresses, and the expressions of five *LcDof* genes were insignificant. In summary, only the class D1 CDF transcription factors exhibited a robust response to low-temperature, drought, and high-temperature stress, indicating that the *LcDof* gene family has evolved class D1 CDF transcription factors that are specifically responsive to diverse abiotic stresses.

This is consistent with recent studies on *CDFs*. For instance, CDF transcription factors can induce the expression of stress-response genes. The A. thaliana AtCDF3 has been shown to regulate the expression of multiple abiotic stress-response genes in plants that respond to extreme temperatures, drought, and osmotic stress [33]. The expression of two *CDF* genes was observed to undergo significant alterations in response to drought and elevated temperatures, with notable changes occurring within the first hour. This suggests that the *LhCDF* genes exhibit a high degree of sensitivity to these two stressors. In summary, the pair of *CDF* genes (*LhDof4* and *LhDof6*) may co-regulate and respond to different abiotic stresses.

### 2.7. qRT-PCR Validation of LcCDFs under Low Temperature and Drought Stress

**Figure 7:**
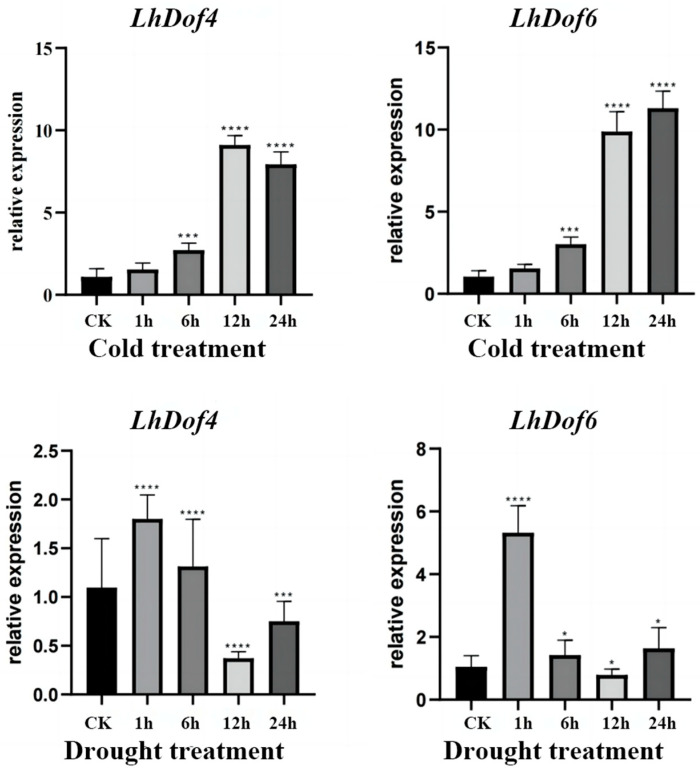
The expression of *LhDof4* and *LhDof6* in cold and drought-treated samples was analyzed by qRT-PCR. Vertical bars represent standard deviation, with 18S serving as the internal reference gene, and each sample was repeated three times. * on the bar chart indicates that the difference between the two control groups reached a significant level, that is, *p* < 0.05, *** means *p* < 0.001, **** means the difference between the two control groups reached a very significant level, that is, *p* < 0.0001.

To verify the accuracy of the abiotic stress transcriptome data for *LcCDFs* (*LhDof4* and *LhDof6*), we conducted qRT-PCR experiments on *Liriodendron* hybrids under cold and drought stress (Figure 7).

Under drought stress, the expression of *LhDof4* and *LhDof6* initially increases, then decreases, and subsequently increases again. The expression levels of both genes drop to their lowest at 12 h, below the control (CK) level, and then increase at 24 h to levels similar to those observed at 1 h. After 1 h, the expression levels significantly increased, then gradually decreased, and finally increased again to return to normal levels. This pattern is consistent with the transcriptome data results under drought conditions. Under low-temperature stress, the expression of *LhDof4* and *LhDof6* gradually increased over time, peaked at 12 h, maintained a high level until 1 day, and then gradually decreased, aligning with the transcriptome data results.

Under low-temperature stress, the expression levels of *LhDof4* and *LhDof6* gradually increased over time, peaking at 12 h. They maintained a high level until 24 h, after which they gradually decreased. This pattern aligns with the transcriptome data results.

### 2.8. LhDof4 and LhDof6 Are Localized to the Cell Nucleus

We conducted a subcellular localization prediction analysis for all *LcDof* genes, including *LcDof4* and *LcDof6*, using an online tool. The results of this analysis are presented in Table 2. Most of the *LcDof* genes are predicted to localize to the nucleus, which is consistent with their roles as transcription factors. However, *LcDof10* and *LcDof11* are predicted to localize in the chloroplast, while *LcDof13* is localized in the mitochondria. All *CDFs* are shown to be localized in the nucleus, likely due to the presence of one or more nuclear localization sequences in the N-terminal structure of CDF proteins. The *LcDof* genes also play regulatory roles in the chloroplasts or mitochondria of plant cells, not just in the nucleus.

**Figure 8:**
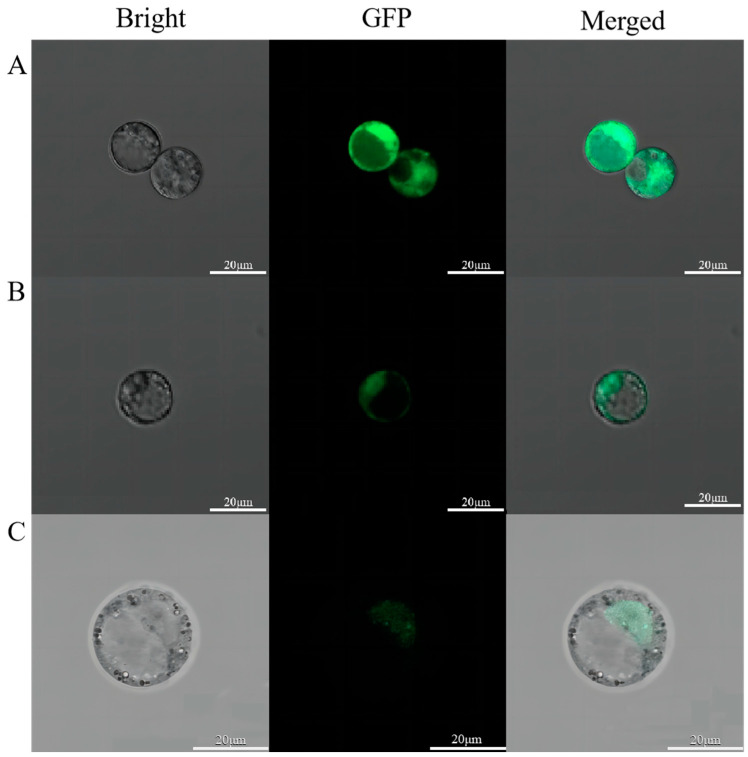
Subcellular localization of *LhDof4* and *LhDof6*. The first panel shows the bright field. The second panel shows green fluorescence (GFP). The third panel is a merged image of the green fluorescence and bright field. (**A**) *p35S:GFP*, (**B**) *p35S:LhDof4-GFP*; (**C**) *p35S:LhDof6-GFP*. Bar = 20 μm.

We then used the cloned *LhDof4* and *LhDof6* sequences to construct fusion fluorescent protein vectors, *p35S:LhDof4-GFP* and *p35S:LhDof6-GFP*, with the unmodified *pJlT166* vector serving as a control. The results showed that in protoplast cells transformed with p35S:GFP, the GFP green fluorescent protein was localized in the nucleus and also distributed in the cell membrane and cytoplasm. In contrast, in protoplast cells transformed with *p35S:LhDof4-GFP* and *p35S:LhDof6-GFP*, the LhDof4-GFP and LhDof6-GFP fusion proteins were primarily localized in the nucleus (Figure 8).

### 2.9. Overexpression of LhDof6 Improved the Cold Tolerance of Liriodendron Hybrid

**Figure 9:**
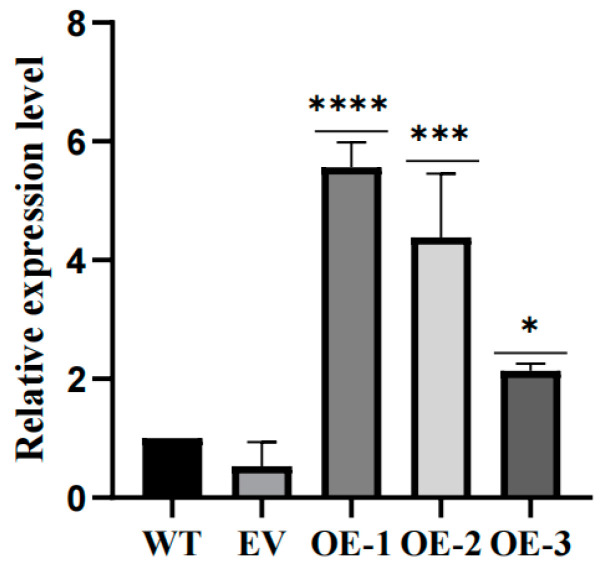
The Relative expression of *LhDof6* in wild-type, no-load and transgenic plants was determined by using 18S rRNA as the reference gene. * on the bar chart indicates that the difference between the two control groups reached a significant level, that is, *p* < 0.05, *** means *p* < 0.001, **** means the difference between the two control groups reached a very significant level, that is, *p* < 0.0001.

Given that our expression analysis indicates that the *LhCDFs* gene may play a role in plant response to cold stress, we further investigated its molecular function in this context. We analyzed the response of *LhDof6* overexpression plants to abiotic stress and characterized their phenotypes. We constructed overexpression vector (Figure S1). Using qRT-PCR, we selected two overexpression lines (Figure 9) and obtained *LhDof6*-OE plants through somatic embryogenesis (SE) technology of *Liriodendron* hybrid. We then compared the freezing stress tolerance of *LhDof6*-OE transgenic plants grown in 1/2 MS medium to that of the wild type. The results indicate that plants overexpressing *LhDof6* exhibit enhanced cold tolerance.

**Figure 10:**
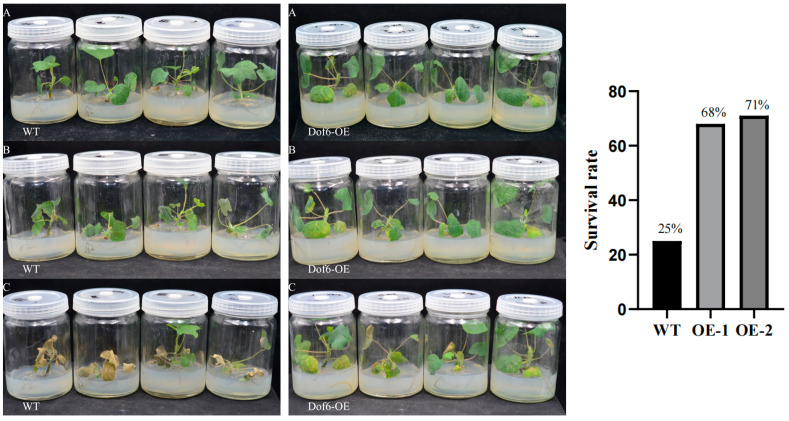
*LhDof6*-OE seedling freeze stress treatment and survival rate statistics. (**A**) Seedlings grown under greenhouse conditions for six months; (**B**) After −20 °C treatment for 30 min followed by 1 d of recovery cultivation; (**C**) After −20 °C treatment for 30 min followed by 7 d of recovery cultivation; Survival rate statistics are displayed on the right.

In each group, 28 similarly grown *Liriodendron* hybrid plants were selected and acclimated at 4 °C (16 h light and 8 h dark) for 7 days. They were then subjected to −20 °C for 20 min. After the treatment, the plants were transferred to a greenhouse at 22 °C for a 7-day recovery period. Photos were taken on the first and seventh days of recovery, and survival rates were calculated on the seventh day. The results showed that the leaves of both the overexpression group and the control group exhibited wilting on the first day of recovery (Figure 10). After 7 days, the leaves of the control group did not recover from wilting; most of them began to wither and die. In contrast, the overexpression group showed significant alleviation of leaf wilting, with leaves resuming spreading and exhibiting a low degree of mortality. Survival rate statistics indicated that the overexpression lines had a survival rate of 68–71%, compared to only 25% in the wild type (Figure 10).

**Figure 11:**
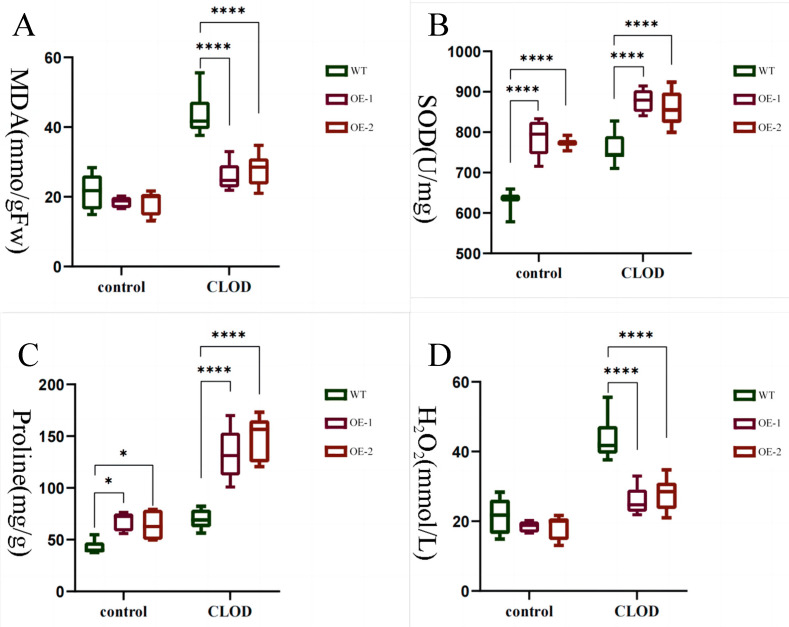
Measurement of various physiological indicators of seedlings treated at −20 °C for 0 and 30 min: (**A**) malondialdehyde (n = 9); (**B**) superoxide dismutase activity (n = 9); (**C**) proline content (n = 9); (**D**) hydrogen peroxide (n = 9). * on the bar chart indicates that the difference between the two control groups reached a significant level, that is, *p* < 0.05; **** means the difference between the two control groups reached a very significant level, that is, *p* < 0.0001.

The occurrence of low temperature stress in plants is widely recognized to induce numerous physiological and metabolic rearrangements, which are mediated by known determinants. Therefore, we conducted measurements on a range of physiological indicators to further elucidate the role of *LhDof6* under freezing stress, as depicted in (Figure 11). After exposure to freezing stress, the MDA content exhibited an increase in both plant types; however, the rise was more pronounced in the wild type, indicating that low temperatures can induce damage to plant cell membranes to some extent, with the wild type experiencing more severe impairment (Figure 11A). Overexpression of *LhDof6* leads to a modest increase in plant proline levels, while under freezing stress conditions, the overexpression lines exhibit significantly enhanced proline accumulation compared to the wild type. This accumulation of proline plays a crucial role in safeguarding plant cells (Figure 11C).

The level of H_2_O_2_ increases rapidly, which can function as a signaling molecule to activate the expression of cold resistance-related genes in plants. However, excessive accumulation of H_2_O_2_ may lead to a spike in intracellular ROS levels and result in oxidative stress. Under normal circumstances, the overexpression of *LhDof6* resulted in an increase in SOD enzyme activity in *Liriodendron* hybrid. Subsequently, exposure to cold temperatures further amplified the SOD enzyme activity, with consistently higher levels observed in the overexpression lines compared to the wild type (Figure 11B). Under freezing stress, wild-type plants exhibited excessive accumulation of H_2_O_2_, which can lead to irreversible damage to the plants (Figure 11D).

The physiological changes observed before and after exposure to cold stress further validate the ability of *LhDof6* to enhance the cold tolerance of *Liriodendron* hybrid seedlings, thereby increasing their survival rate in extreme environments. These analyses underscore the potential beneficial role played by the *LhDof6* gene in facilitating plant adaptation to cold stress.

## 3. Discussion

The Dof transcription factors possess a highly conserved single-finger domain, known as a zinc finger domain, consisting of 52 amino acid residues. This specific domain facilitates precise binding to DNA [34]. In this study, we identified 17 *Dof* genes in the *L. chinense* genome, designated as *LcDof1~17* based on their chromosomal location. The number of *LcDof* genes is relatively small compared to some higher angiosperms, i.e., *Arabidopsis thaliana* (36), *Oryza sativa* (30), *Sorghum bicolor* (28), *Glycine max* (78), *Hordeum vulgare* (21), *Nicotiana tobacum* (17) and *Populus trichocarpa* (43) [35]. The findings suggest a potential association between the number of *Dof* gene family members and their evolutionary states as well as patterns of family expansion.

The protein length, molecular weight (MW), and isoelectric point (pI) of LcDof proteins exhibit significant variations among gene families, indicating their structural diversity and potential adaptation to diverse environments. These characteristics suggest that *LcDofs* may possess distinct biological regulatory functions in different environments or conditions, particularly under abiotic stress. Furthermore, the variability in length, molecular weight, and isoelectric point of Dof proteins across species implies divergent biological functions of *Dof* genes between different organisms. This divergence may be attributed to differences in physicochemical properties and spatial structures of these proteins.

The collinearity analysis revealed six pairs of gene duplication events in the *LcDof* genes, with only one pair attributed to tandem duplication, while the remaining pairs were a result of segmental duplication. The fact that all duplicated genes belonged to the same category suggests that *L. chinense* has undergone at least one whole-genome duplication event since its divergence. The chromosomal localization analysis of the *LcDof* genes revealed an uneven distribution pattern, with most genes located at the terminal regions of 11 chromosomes and no more than two genes per chromosome. This spatial arrangement suggests active expression of the *Dof* gene family [28]. The involvement of several transcription factors (TFs) in plant stress responses has been identified, with some participating in intricate regulatory networks. These TFs are predominantly encoded by polygenic families that have undergone multiple rounds of gene replication throughout the evolution of land plants [36,37,38]. The clustered distribution of *LcDof* genes at the chromosome termini may be associated with the typically more accessible chromatin structure in these regions, which could facilitate active gene expression. Conversely, the chromatin structure near the centromere is generally more compacted, potentially constraining gene expression [39].

The regulation of plant hormones is achieved through the binding and coordinated interaction of various transcription factors with cis-acting elements present in the promoters of plant hormone response genes [40]. The Dof proteins function as regulators of plant hormone response genes and have been demonstrated to mediate the gibberellin response [41]. The Dof transcription factor also exhibits circadian rhythms and plays a pivotal role in perceiving plant photoperiods and regulating flowering time. Analysis of *JcDof1* and *JcDof3* in *Jatropha curcas* seedlings revealed their expression patterns under long-day, short-day, and continuous light conditions, as well as their interaction with F-box proteins to modulate photoperiodic flowering [42]. The cis-elements present in the promoters of *LcDof* genes predominantly consist of light-responsive elements, plant hormone regulatory elements, stress-related elements, and growth and development regulatory elements. This implies that the *LcDof* genes exert comparable effects on photoperiodic response, abiotic stress response, and plant hormones. Temporal transcriptome analysis of *Liriodendron* hybrid under low-temperature, high-temperature, and drought conditions revealed that several *LcDof* genes exhibited a transcriptional profile characterized by an initial upregulation followed by downregulation in response to low-temperature conditions. Notably, the expression dynamics of *LhDof4* and *LhDof6* were particularly remarkable. Quantitative Real-Time PCR (qRT-PCR) analysis demonstrated that the expression levels of *LhDof4* and *LhDof6* increased approximately tenfold within 12 to 24 h after exposure to low temperatures compared to their pre-treatment levels. This strongly suggests that these two genes may play a pivotal role in the physiological response of *Liriodendron* hybrid to low temperatures.

The Cycling Dof Factor (*CDF*) is capable of regulating various aspects of plant growth and development, including the photoperiodic control of flowering as well as root and shoot growth. While most functional characteristics of *CDFs* have been extensively studied in *Arabidopsis*, recent data indicate that their diverse roles also extend to other plant species [42,43,44,45]. The role of DOF transcription factors (TFs) has been extensively investigated in numerous plant species, including important crops such as maize, wheat, rice, potato, and bananas, in response to various environmental stress conditions [46,47]. Transcriptomic analysis has revealed a limited overlap in the stress response genes regulated by *GI* and *CDF3*, indicating that these two proteins have distinct functions specifically under low temperature and osmotic stress conditions [44]. Further comprehensive and functional analyses are imperative to elucidate the precise roles of these factors in plant responses to diverse environmental stress conditions. Moreover, it has been reported that overexpressing *AtCDF3* or *SlCDF3* in *Solanum lycopersicum* enhances tolerance to salt stress [33]. These reports suggest that *SlCDFs* may have a crucial regulatory function in the upstream pathways of salinity and drought response, similar to their counterparts in *Arabidopsis*. Furthermore, the overexpression of *LhDof6* significantly mitigated the mortality rate of *Liriodendron* hybrid seedlings exposed to extreme temperatures as low as −20 °C. Physiological changes observed before and after cold stress exposure also provide evidence that *LhDof6* can enhance the cold tolerance of *Liriodendron* hybrid seedlings, thereby increasing their survival rate in harsh environments. These analyses indicate an active role for the *LhDof6* gene in facilitating plant adaptation to low temperature stress.

## 4. Materials and Methods

### 4.1. Identification of Dof Gene in L. chinense

To identify the *Dof* gene in *L. chinense*, 37 typical and atypical Dof protein sequences of *Arabidopsis thaliana* were downloaded from the TAIR database (https://www.arabidopsis.org/Blast/index.jsp, accessed on 21 November 2021). From the pfam website (http://pfam-legacy.xfam.org/, accessed on 22 November 2021), the Dof hidden Markov number is PF02701, and this number has been used as the query condition. Blastp and HMMER were used to query the target sequence in the L. chinense protein database, and candidate sequences were obtained. Based on the Dof conserved domains, CDD-search (https://www.ncbi.nlm.nih.gov/Structure/cdd/wrpsb.cgi, accessed on 22 November 2021) was used to check the conserved domains of candidate sequences to further screen out redundant sequences, and finally obtain the target sequence. Gene properties, including length, molecular weight, and isoelectric point of each protein, were determined using the ExPASy website (https://web.expasy.org/protparam, accessed on 3 January 2022) tool. Subcellular localization of *LcDof* genes was predicted by Cell-PLoc 2.0 (www.csbio.sjtu.edu.cn/bioinf/Cell-PLoc-2, accessed on 3 January 2022).

### 4.2. Phylogenetic Analysis and Conserved Domains and Gene Structure Analysis

*Arabidopsis thaliana*, *Amborella trichopoda*, *Zea mays*, and *Oryza sativa* Dof protein sequences used to construct the phylogenetic tree were downloaded from Phytozome (https://phytozome-next.jgi.doe.gov, accessed on 3 January 2022). Multiple sequence alignment of *Dof* gene family members was performed using MAFFT software (https://mafft.cbrc.jp/alignment/software, accessed on 25 January 2022) with default parameters. MAGE 7.0 was utilized to construct the phylogenetic tree, employing the neighbor-joining method with a bootstrap value of 1000 to analyze the evolution of the *Dof* gene in *L. chinense*.

The gene structure information of each *LhDof6* gene was acquired from the genomic feature file (GFF3) and displayed using Tbtools software (https://github.com/CJ-Chen/TBtools/releases, accessed on 12 February 2022), while the chromosomal location and microsynteny of *LhDof6* were visualized using the Tbtools software. The Multiple Collinearity Scan toolkit (MCScanX) program was used to verify putative paralogous genes (blast hits E-value cutoff < 1 × 10^−6^, collinearity >  70%). The cis-acting elements of *LhDof6* genes were analyzed by PlantCARE (http://bioinformatics.psb.ugent.be/webtools/plantcare/html/, accessed on 15 February 2022) and displayed using TBtool software. The conserved motifs of LcDof proteins were predicted using MEME (v5.4.1) (https://meme-suite.org/meme/tools/meme, accessed on 21 February 2022) with the following settings: the discovery mode was classic, site distribution was zero or one occurrence per sequence, the background is 0-order background model, the maximum number of different motifs: 20, minimum motif width: 6, and maximum motif width: 50, and displayed using the TBtool software.

### 4.3. Plant Materials and Genetic Transformation of Liriodendron Hybrid

*Liriodendron* hybrid seedlings generated through somatic embryogenesis (SE) were used as the starting material throughout this study [48]. Before any experiments were performed, plantlets were taken out of the culture medium vessel and acclimatized in a greenhouse for 2 weeks (22 °C, long day photoperiod of 16 h light/8 h dark and 75% relative humidity). For various abiotic stress treatments, plants were transferred to a growth chamber (long time photoperiod of 16 h light/8 h dark and 75% relative humidity): to simulate cold or heat or drought stress, plantlets were subjected to a 4 °C or 15% PEG6000 treatment, respectively, for 1 h, 6 h, 12 h and 1 d in the growth chamber [49].

The full-length coding sequence (CDSs) of *LhDof6* was amplified from *Liriodendron* hybrid by PCR, and cloned into the pBI121 vector with overexpression of *LhDof6* (hereinafter referred to as *LhDof6*-OE) under the control of CaMV 35S promoter and ScaI and XbalI, respectively. After the vector was constructed, the vector was transformed into *Agrobacterium* receptive cell EHA105, and the monoclonal colony was selected to verify its correctness before transfection [48]. Positive callus was obtained through genomycin screening, and mutants identified via PCR and sequencing were used for subsequent experiments.

### 4.4. RNA Extraction and qRT-PCR Analysis

Based on the transcriptome data of *Liriodendron* hybrid under different abiotic stresses, a heatmap of *LhDof6* gene expression was generated using Tbtools. The transcriptome data used in this study has been archived and can also be obtained on the NCBI website, cold and heat stress accession numbers were PRJNA679089 (https://www.ncbi.nlm.nih.gov/bioproject/PRJNA679089/, accessed on 19 June 2022), and drought stress accession number was PRJNA679101 (https://www.ncbi.nlm.nih.gov/bioproject/PRJNA679101/, accessed on 19 June 2022).

RNA degradation and contamination were monitored on 1% agarose gel. RNA purity was detected using the NanoPhotometer^®^ spectrophotometer (IMPLEN, Westlake Village, CA, USA). Using the Bioanalyzer 2100 system (Agilent Technologies, Santa Clara, CA, USA), The RNA Nano 6000 Assay Kit (Agilent Technologies, CA, USA) assesses RNA integrity and synthesizes cDNA using the HiScript^®^ III 1st Strand cDNA Synthesis Kit (Vazyme, Nanjing, China), using the extracted RNA as a template. Using primer3 website (https://www.yeastgenome.org/primer3, accessed on 30 June 2022) design quantitative expression *LcDofs* primers, RT-qPCR using Roche Lightcyler^®^ 480 instrument II, use 2× AceQ^®^ qPCR SYBR^®^ Green Master Mix (Without ROX) (Vazyme, Nanjing, China). The PCR mixture consists of 2× AceQ^®^ qPCR SYBR^®^ Green Master mix (Without ROX) 10 μL, each primer 0.4 μL, cDNA template 1 μL (10 ng/μL), 8.2 μL ddH_2_O added, the final volume was 20 μL. The internal reference gene was *L. chinense* 18S gene. The reaction process is as follows: 95 °C—10 min, 95 °C—10 s, 57 °C—30 s, 40 cycles. All reactions were performed in 96-well plates. Biological replicates were performed for each reaction, as well as three technical replicates. All data generated by real-time PCR amplification were analyzed by 2^−ΔΔCT^ [50].

### 4.5. Subcellular Localization

To verify the subcellular localisation of *LhDof4* and *LhDof6*, we obtained the *LcDof4* and *LcDof6* target fragment by PCR using the *LhDof4* and *LhDof6* sequence as the reference sequence and the cDNA of *Liriodendron* hybrid seedling as the template. The plasmid *pJIT166-GFP* was digested with XbaI and BamHI enzymes, and the linear vector fragment was ligated with the target gene fragment to construct the *p35S:LhDof4/6-GFP* fusion expression vector. Plasmids were extracted using an endotoxin-free plasmid extraction kit (TIANGEN, Beijing, China). The callus of *Liriodendron* hybrid cultured for 20 days was used to prepare protoplasts, and protoplasts were slowly and gently dissolved into a solution (10 mL) containing 0.5 M mannitol, 20 mM MES, pH 5.7, 20 mM KCl, 0.1% (*w*/*v*) bovine serum albumin, 10 mM CaCl_2_ and digested at 28  °C under dark conditions for 3 h [51], the protoplasts were transformed by PEG6000, pipetted into a 6-well cell culture plate, and cultured at 23 °C under dark for 16~48 h [51], then the fluorescence effect of protoplasts was observed by ZEISS LSM 800 fluorescence microscope (Carl Zeiss, Oberkochen, Germany).

### 4.6. p35S: LhDof6 Positive Seedlings and Cold Stress Tolerance Assay

*LhDof6*-OE seedlings generated through SE. Each group selected 28 uniformly growing and healthy 6-month-old *Liriodendron* hybrid seedlings for a freeze tolerance test. The seedlings were exposed to −20 °C for 20 min and then transferred to a greenhouse environment (26 °C with 16 h of light/8 h of darkness) for observation. After the treatment, the plant leaves were washed with distilled water, dried with paper towels, weighed, and then flash-frozen in liquid nitrogen before being stored at −80 °C for future use. The assay kits for hydrogen peroxide (H_2_O_2_), lipid peroxidation malondialdehyde (MDA), proline (PRO), and superoxide dismutase activity (SOD) were purchased from Nanjing Jiancheng Bioengineering Institute, Jiangsu provenience, China.

### 4.7. Data Analysis

Data were plotted using Prism 8.0 software, and the significance of differences between samples was assessed using *t*-tests and one-way ANOVA. * on the bar chart indicates that the difference between the two control groups reached a significant level, that is, *p* < 0.05; ** means *p* < 0.01, *** means *p* < 0.001, **** means the difference between the two control groups reached a very significant level, that is, *p* < 0.0001. The content of malondialdehyde, superoxide dismutase activity, proline content and hydrogen peroxide in plants before and after freezing stress were significantly analyzed.

## 5. Conclusions

Our research findings indicate that the *L. chinense* genome harbors a total of 17 *Dof* genes. The physicochemical properties of the proteins encoded by these genes exhibit considerable variation. Specifically, the protein lengths span from 160 to 635 amino acids, while their molecular weights range between 17.01 and 71.60 kDa. The majority of LcDof proteins are categorized as unstable, rendering them susceptible to alterations caused by external environmental factors. The DOF domains within the *LcDof* gene family exhibit complete conservation. The six clades are characterized by distinct motif structures and gene architectures, implying potential functional similarities among *LcDof* genes within each clade. Phylogenetic analysis revealed that the *Dof* genes can be classified into eight distinct subfamilies, with the notable absence of *LcDof* genes in the C3 subfamily. Collinearity analysis suggests that segmental duplications are primarily responsible for the expansion of the *LcDof* gene family, which underwent five such events along with one tandem duplication event. Analysis of promoter regions also indicates a rich presence of cis-acting elements. The elements are classified into four categories: growth and development-related elements, light-responsive elements, abiotic stress-responsive elements, and plant hormone-responsive elements. Moreover, there is a relatively abundant presence of abiotic stress-responsive elements and plant hormone-responsive elements. The transcriptome data of *Liriodendron* hybrid under various abiotic stresses reveals a high expression level of *Dof* genes from the D1 subfamily, particularly highlighting the significant responses of *LcDof4* and *LcDof6*. This finding was further confirmed through RT-qPCR analysis, suggesting that *LcDof4* and *LcDof6* may play a crucial role in positively regulating the response to cold stress. We successfully cloned the *LcDof4* and *LcDof6* genes, and observed their expression in the cell nuclei of protoplasts derived from *Liriodendron* hybrid callus. Furthermore, we conducted overexpression experiments with *LhDof6* in *Liriodendron* hybrid, which resulted in a significant increase in the survival rate of six-month-old seedlings under −20 °C conditions. Furthermore, a series of physiological measurements have confirmed that the overexpression of *LhDof6* significantly enhances the plant’s cold tolerance. In summary, we have successfully identified and analyzed the *Dof* gene family in *L. chinense* for the first time, screened for genes associated with cold tolerance, and conducted an initial functional analysis on the role of *LhDof6* in enhancing cold tolerance.

## Figures and Tables

**Table 1:** *LcDof* genes and their related information. (Len: protein length; MW: molecular weight; PI: isoelectric point; AI: aliphatic index; II: Instability index).

Name	Gene ID	Chromosome Position	Len	MW (kDa)	PI	AI	II	Stability
*LcDof1*	Lchi13427	chr1:14905449:14907434	315	34,392.72	9.37	44.32	85.81	unstable
*LcDof2*	Lchi13426	chr1:149362541:4938133	341	37,100.66	7.61	64.99	56.63	unstable
*LcDof3*	Lchi14379	chr2:3910385:3912566	307	33,571.54	6.06	53.97	45.93	unstable
*LcDof4*	Lchi02891	chr2:93446246:93449976	493	53,973.3	5.82	50.91	54.73	unstable
*LcDof5*	Lchi18955	chr4:13910965:13912129	160	17,009.23	9.56	49.5	68.83	unstable
*LcDof6*	Lchi14330	chr4:101466704:101470296	489	53,203.72	6.05	53.17	58.92	unstable
*LcDof7*	Lchi21078	chr7:41859297:41862848	512	55,042.32	5.7	52.42	44.4	unstable
*LcDof8*	Lchi11874	chr8:60674389:60680714	340	36,995.94	8.86	52.53	59.23	unstable
*LcDof9*	Lchi05004	chr11:47813134:47836555	357	39,425.93	8.36	55.71	61.17	unstable
*LcDof10*	Lchi07493	chr12:69278241:69290309	635	71,607.42	6.93	78.44	45.06	unstable
*LcDof11*	Lchi08966	chr13:3769782:3770546	225	24,352.18	7.62	57.16	38.83	stable
*LcDof12*	Lchi12994	chr13:4230878:4232196	321	34,753.39	8.44	63.52	52.58	unstable
*LcDof13*	Lchi19181	chr15:1327552:1332351	434	47,497.9	6.89	62.51	41.14	unstable
*LcDof14*	Lchi13915	chr16:27159341:27160497	322	35,211.79	9.17	55.43	54.53	unstable
*LcDof15*	Lchi13929	chr16:27411223:27412002	232	24,380.43	8.25	64.7	59.88	unstable
*LcDof16*	Lchi21177	chr18:12284594:12285747	332	35,675.98	9.07	51.14	59.25	unstable
*LcDof17*	Lchi24080	chr18:43150014:43164244	298	32,866.91	8.00	57.95	55.84	unstable

**Table 2:** Subcellular localization prediction results for *LcDof* genes.

Gene	Predicted Position
*LcDof1*	nucleus
*LcDof2*	nucleus
*LcDof3*	nucleus
*LcDof4*	nucleus
*LcDof5*	nucleus
*LcDof6*	nucleus
*LcDof7*	nucleus
*LcDof8*	nucleus
*LcDof9*	nucleus
*LcDof10*	chloroplast
*LcDof11*	chloroplast
*LcDof12*	nucleus
*LcDof13*	mitochondrion
*LcDof14*	nucleus
*LcDof15*	nucleus
*LcDof16*	nucleus
*LcDof17*	nucleus

## Data Availability

The data and results are available to every reader upon reasonable request.

## Supplementary Materials

The following supporting information can be downloaded at: https://www.mdpi.com/article/10.3390/plants13142009/s1, Figure S1: Construction and verification of *LhDof6-OE*, *35S-LhDof4-GFP* and *35S-LhDof6-GFP* expression vector; Table S1: Primer Sequence.
