# Supplementary material for: The Role of Liriodendron Dof Gene Family in Abiotic Stress Response
Source: Plants (Basel). 2024 Jul 22;13(14):2009. doi: 10.3390/plants13142009 (PMC11281171; doi:10.3390/plants13142009)
Supplement: Supplementary file 1 [file plants-13-02009-s001.zip › Supplementary Materials.pptx]

## Slide 1
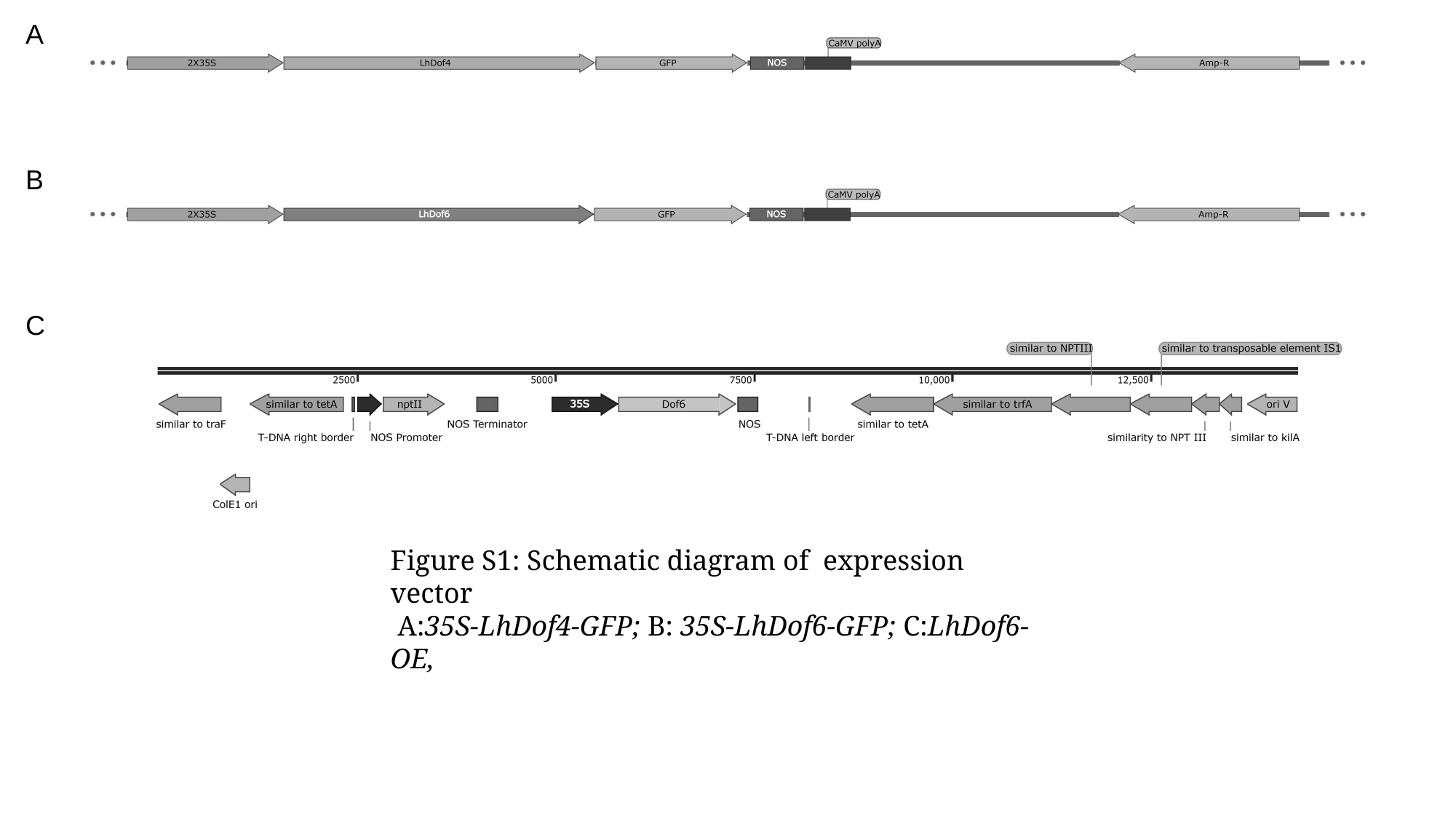

A
B
C
Figure S1: Schematic diagram of expression vector
 A:35S-LhDof4-GFP; B: 35S-LhDof6-GFP; C:LhDof6-OE,

## Slide 2
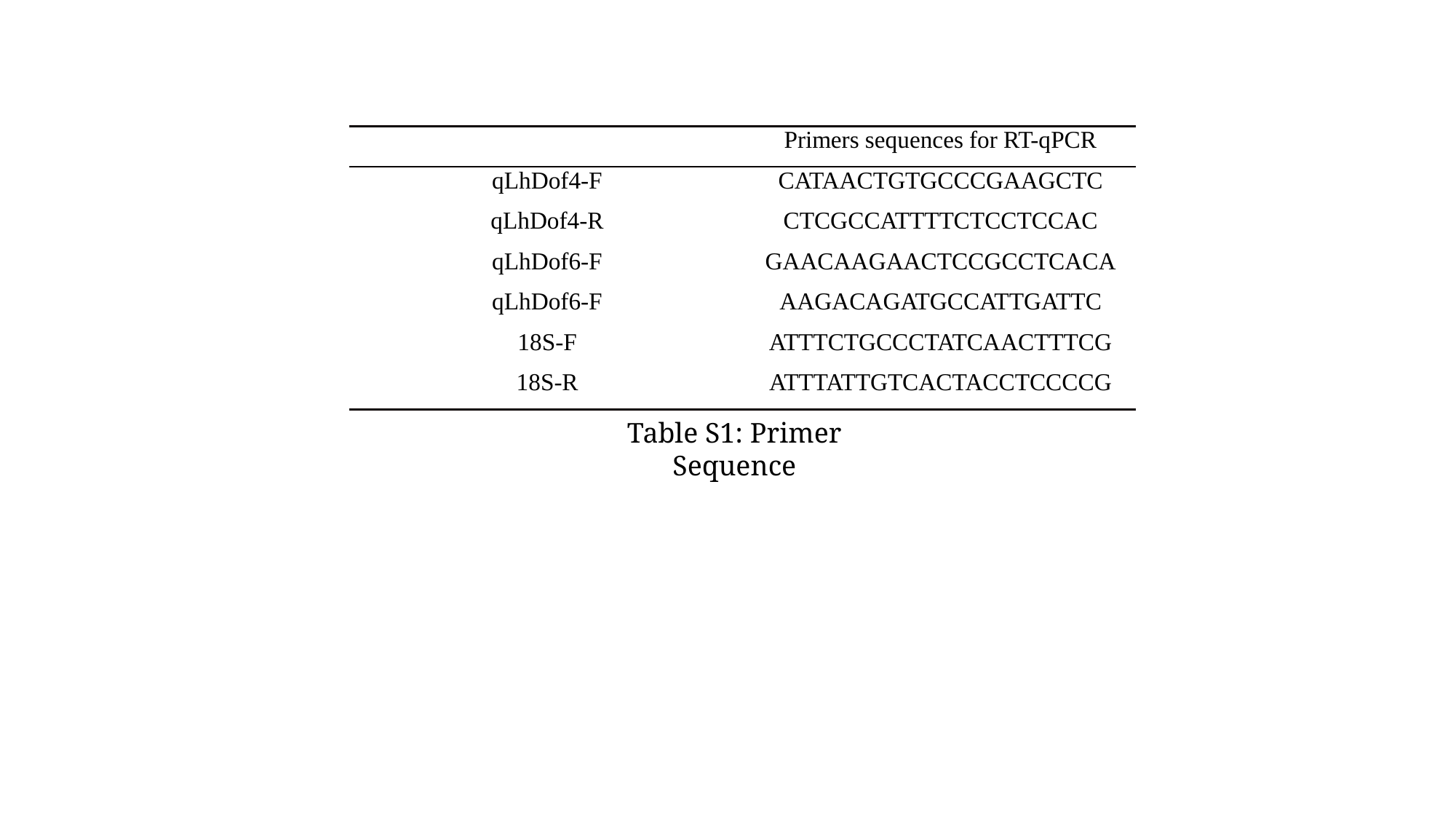

| | Primers sequences for RT-qPCR |
| --- | --- |
| qLhDof4-F | CATAACTGTGCCCGAAGCTC |
| qLhDof4-R | CTCGCCATTTTCTCCTCCAC |
| qLhDof6-F | GAACAAGAACTCCGCCTCACA |
| qLhDof6-F | AAGACAGATGCCATTGATTC |
| 18S-F | ATTTCTGCCCTATCAACTTTCG |
| 18S-R | ATTTATTGTCACTACCTCCCCG |
Table S1: Primer Sequence
